# Tumor suppressor BLU promotes TRAIL-induced apoptosis by downregulating NF-κB signaling in nasopharyngeal carcinoma

**DOI:** 10.18632/oncotarget.14126

**Published:** 2016-12-23

**Authors:** Jiahui Zhou, Zunnan Huang, Ziyou Wang, Shumin Liu, Alf Grandien, Ingemar Ernberg, Zhiwei He, Xiangning Zhang

**Affiliations:** ^1^ Department of Pathophysiology and China-America Cancer Research Institute, Guangdong Provincial Key Laboratory of Medical Molecular Diagnostics, Dongguan Scientific Research Center, Guangdong Medical University, Dongguan, Guangdong, China; ^2^ Department of Microbiology, Tumor and Cell Biology, Karolinska Institutet, Stockholm, Sweden; ^3^ Center for Haematology and Regenerative Medicine, Karolinska Institutet, Stockholm, Sweden

**Keywords:** nasopharyngeal carcinoma, tumor suppressor gene, BLU/ZMYND10, TRAIL, NF-κB

## Abstract

A putative tumor suppressor *BLU* mapped on the chromosomal 3p21 region, is frequently lost in human tumors including nasopharyngeal carcinoma (NPC). To explore the underlying mechanism of tumor suppression by *BLU*, its potential to promote apoptosis induced by TRAIL, an effector molecule elaborated by natural killer-T (NKT) cells was investigated. *BLU* was re-expressed in NPC-derived HNE1 cells by recombinant adenoviral infection and the cells were challenged with recombinant TRAIL. The growth inhibition of *BLU* was assayed and apoptosis was examined by flow cytometry-based tetramethylrhodamine ethyl ester (TMRE) and annexin V staining, cleavage of pro-caspase-8 and poly ADP ribose polymerase (PARP). The modulation of NF-κB pathway by *BLU* was evaluated by the reporter activity and estimation of the level of the molecules involved such as IKKalpha, p65 NF-κB, as well as NF-κB induced anti-apoptotic factors cFLIP_L_ and cIAP2. The expression of *BLU* exerted in vitro and in vivo growth inhibitory effect and promoted TRAIL-induced apoptosis. This phenomenon was validated by FACS-based assays of mitochondrial membrane potential (BLU vs. Vector 87.8% ± 7.7% and 72.1%±6.7% at 6h exposure to TRAIL) and phosphatidylserine turnover (*BLU* vs. vector: 28.7%±2.9% and 22.6%±2.5%), as well as, enhanced caspapse-8 cleavage. Similar with the findings that *BLU* promotes chemotherapeutic agent-induced apoptosis, it also augmented death receptor-induced pathway through NF-κB pathway inhibition. In conclusion, *BLU* suppressed tumor formation by strengthening the antitumor immunity.

## INTRODUCTION

Nasopharyngeal carcinoma (NPC) is a head and neck cancer arising from the epithelial cells in the lining of nasopharynx [1]. The prevalence is distinctively manifested geographically and ethnically, with a high incidence in some parts of the world, including southern China, Southeast Asia, northern Africa, Alaska, and Greenland [2]. In the endemic regions, the incidence of 25 cases per 100,000 population has been recorded annually, which is about 25-fold higher than the rest of the world [3–5]. The differences in the geographical and racial distribution indicate the multifactorial etiology of the particular tumor; these include environmental factors, food consumption, the infection of Epstein-Barr virus (EBV), a lymphotropic human herpesvirus, and genetic susceptibility. This remarkable distribution of NPC indicates that the development of nasopharyngeal cancer may be associated with the genetic and environmental factors [3].

Hitherto, radiotherapy and concurrent chemoradiotherapy are the primary treatments of primary NPC [6]. The tumor cells of NPC are sensitive to radiotherapy; however, the use of radiotherapy, chemoradiotherapy, or targeted radiotherapy has not significantly improved the survival rate of the patients. An overall locoregional failure after the initial treatment is approximately 10% [7]. The regional lymph node, distant metastasis, and loco-regional recurrence are the major indications of the therapeutic failure of NPC [8–10]. Elucidation of the underlying mechanism of NPC genesis is necessitated in order to improve the efficacy of the therapy.

Apoptosis is a host mechanism to eliminate the unwanted cell clones and serve as the end effect of antitumor immune surveillance. The extrinsic or the death receptor pathway contributes to the removal of the mutant or transformed cells; the pathway is activated by death ligands elaborated by the effector cells in antitumor immunity, cytotoxic T lymphocytes (CTL), and natural killer (NK) cells.

NF-κB is a transcription factor, a heterodimer of different subunits, and is implicated in immune response, inflammation, and carcinogenesis [11]. The subunits belong to a protein family comprising of RelA (p65), RelB, c-Rel, p50 (p105 precursor), and p52 (p100 precursor) [12]. The subunits form homo- or heterodimeric complexes with each other and also with their inhibitory proteins (I-κB proteins) [11]. In the quiescent physiologic state, NF-κB is sequestered in the cytoplasm in an inactive form and associated with I-κB proteins like, IkI- κB a or the precursor proteins, p100 and p105. The I-κBs molecules are phosphorylated by I-κB B kinases (IKKs) before they are dissociated from the complex. They are ubiquitinated after the dissociation, followed by degradation. The degradation of I-κB from the NF-κB : I-κB complex is an essential step for activating the released NF-κB [13–15]. It binds to specific DNA sequences on the regulatory elements upstream of the coding region of a gene, through the Rel homology region.

NF-κB transcriptionally activates anti-apoptotic genes that function in the death-inducing signaling complex (DISC) as well as in the mitochondria. These anti-apoptotic genes, such as *IAP-1*, *IAP-2*, *c-FLIP*, *TRAF1*, and *TRAF2*, coordinate their inhibitory effects to interfere with the receptor-mediated death at the DISC. IAP-1 and IAP-2 can interact directly with caspases to block their activation, thereby facilitating their degradation [16].

As a cytotoxic cytokine in this category, TRAIL can kill tumor cells in vivo without harming the normal tissues [17]. TRAIL binds to death receptor 4 (DR4)/TRAIL-R1 and DR5/TRAIL-R2, which are mainly expressed on transformed cells and triggers apoptotic signal initiated by the cleavage of caspase-8. In contrast to tumor cells, non-neoplastic cells tend to express the TRAIL decoy receptors DR3/TRAIL-R3 and DR6/TRAIL-R4; the host cells are rendered less sensitive to TRAIL-mediated death. [18, 19]. The finding has initiated the development of TRAIL receptor (TRAIL-R) agonists as an anticancer drug in clinical therapy. This hypothesis is further supported by the finding that malignant cells could resist TRAIL-mediated cytotoxicity, mainly because of the silencing of tumor suppressor genes that downregulate anti-apoptotic signaling pathways [20].

Tumor suppressor genes (TSGs) are frequently lost in malignancies. With the loss-of-function of these TSGs, the cells are no longer harnessed in growth and proliferation by regulation of the cell cycle entry, thus, triggering apoptosis [21, 22].

*BLU* encodes a protein of the family that possesses a motif of zinc finger myelogenous Nervy domain (ZMYND). Some of the proteins, like ETO/ZMYND3, are implicated in the genesis of malignancies through protein-protein interactions, as repressors of transcription. A fused protein, AML, comprising of ETO and RUNX, is formed due to the chromosomal translocation t(8:21) in the genesis of acute myelogenous leukemia. ETO of the ZMYND family associates with histone deacetylase (HDAC) to suppress the transformation potential of oncogene *RUNX2* [23].

*BLU* is highly expressed in many normal tissue types including lung, testes, and the uterine cervix. However, it is downregulated in lung, breast, kidney, neuroblastoma, and esophageal squamous cell carcinoma [24–27]. It is also frequently observed to be hypermethylated in primary tumors [28–31]. The structural features of the *BLU* protein involve the C-terminal region encompassing the MYND domain. The domain consists of several cysteine and histidine amino acid residues, which may be involved in protein–protein interactions to repress gene transcription

The tumor suppression of *BLU* has been indicated by its frequent loss in some human malignancies, but the underlying mechanisms are largely unknown. Previously, we have shown that similar to the tumor suppressors RASSF1A [32] and NGDR 2 [33], *BLU* down-regulates JNK signaling and inhibits the promoter activity of cyclin D1, thereby reducing its expression level to arrest the cell cycle in G1 phase [34]. The inhibition of cell cycle entry contributes to the proliferation inhibition of *BLU*. Park et al. have shown that *BLU* potentiated apoptosis induced by the chemotherapeutic agent, paclitaxel to exert tumor suppression [35]. Promotion of paclitaxel induced by BLU correlated with enhanced activities of pro-apoptotic molecules Bax and p21, and inactivation of Bclx_L_ and NF-κB inducing Bcl-2, both are anti-apoptotic.

In the present study, we explored whether *BLU* inhibited the NF-κB pathway, and whether the NF-κB-dependent factors cFLIP and cIAP2 promoted death receptor-induced apoptosis.

## RESULTS

### Growth inhibitory effects on HNE1 cells exerted in vitro and in vivo by BLU

**Figure 1:**
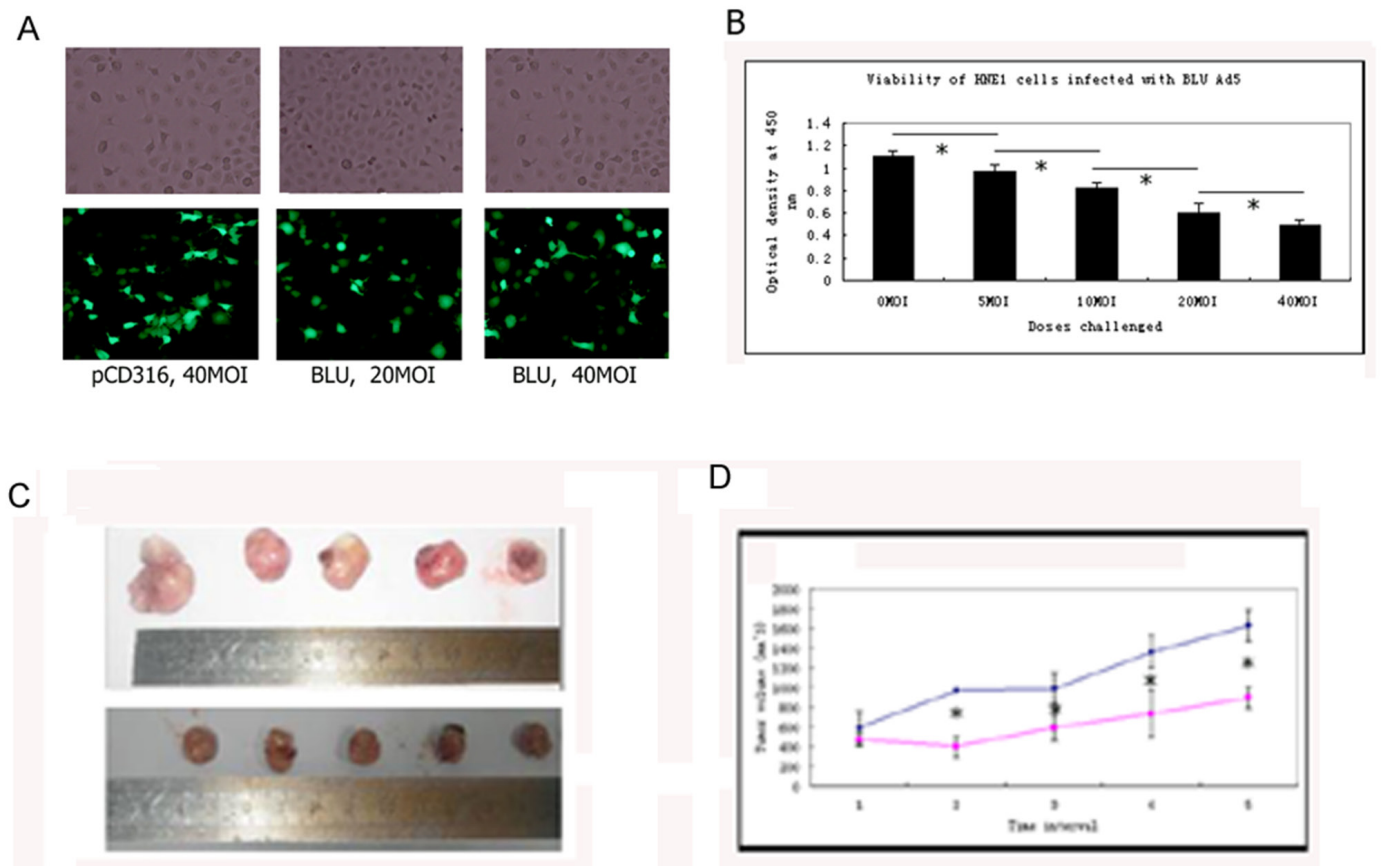
Growth inhibitory effects on HNE1 cells exerted in vitro and in vivo by *BLU* **A**. Re-expression of BLU was confirmed the presence of green fluorescence viewed with microscopy. **B**. Growth inhibitory effect of *BLU* on HNE1 cells was measured with CCK8 kit. Significant differences was suggested by P<0.05 depicted with an asterisk (*). **C**. Growth of xenografted HNE1 cells was inhibited by *BLU* in Balb/C mice. Two weeks after subcutaneously inoculation, palpable tumors were identified. Recombinant *BLU* Ad5 and the control adenovirus with pCD316 were injected intratumorally. The long and short radiuses were measured with caliber every other day. The mice were sacrificed after two-week observation. The tumors were harvested by surgical removal and photographed (C); Upper panel: control group with injection of pCD316 Ad5; lower panel: experimental group with injection of *BLU* Ad5; **D**. The tumor volumes were calculated according to the formula v=ab^2^/2; a and b were the long and short radiuses mentioned above. The growth curve was plotted according to the tumor size registered every other day. Data derived from tests with three groups each containing 6 mice and presented as mean ± SD. The asterisk (*) in D depicted p< 0.05 in statistics.

The expression of EGFP-tagged *BLU* in HNE1 cells infected with pCD316-Ad5, and 20 and 40 MOI BLU-Ad5 was confirmed by green fluorescent signals (Figure 1A). After infection with 0, 5, 10, 20, and 40 MOI BLU-Ad5, the viability of the cells assayed using the CCK8 kit was found to be decreased in a dose-dependent manner, with a significant difference at each dose (Figure 1B), suggesting that BLU possessed an inhibitory effect on the cell proliferation. The viability assay revealed that the recombinant pCD316 adenovirus also presented cytotoxicity albeit much weaker (data not shown). When intratumorally injected into human NPC xenografts in nude mice, the growth of the tumors was remarkably inhibited by recombinant BLU-Ad5 (Figure 1C and 1D).

### TRAIL induced apoptosis in HNE1 cells was potentiated by expression of BLU

**Figure 2:**
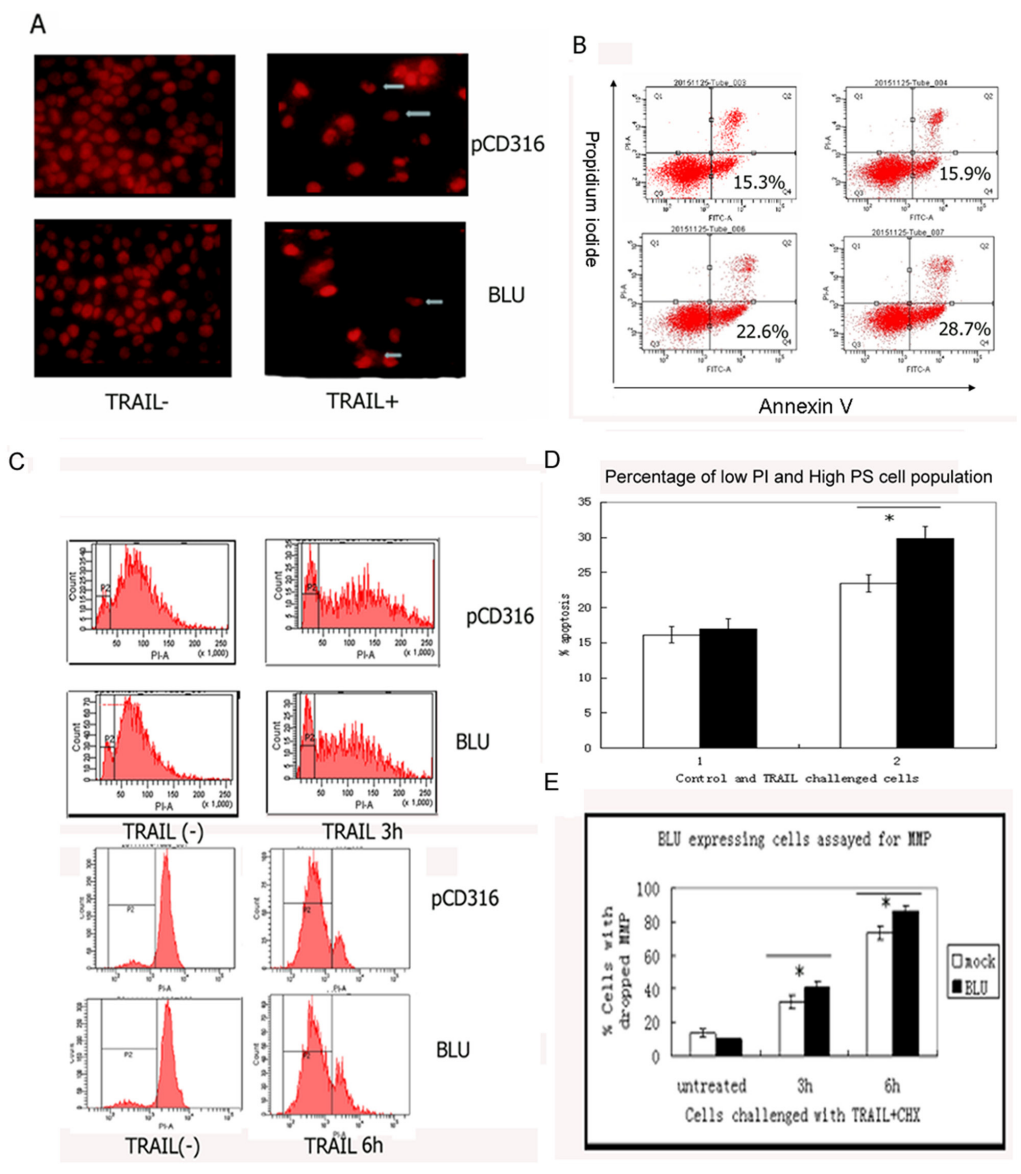
TRAIL induced apoptosis in HNE1 cells was potentiated by expression of *BLU* **A**. The morphology of TRAIL treated HNE1 cells. The cells were co-incubated with 200 ng/ml TRAIL, then were collected and stained with PI, and viewed with fluorescence microscopy. Apoptosis was confirmed by the fragmented nuclei and reduced cell numbers. **B**. Turnover of surface phosphatidylserine (PS) on TRAIL treated HNE1 cells. The cells grown in 6 well culture plates were treated as above. They were harvested, stained with annexin V kit and analyzed with a FACScan. The quadrant 4 (Q4) with low PI and high PS represented apoptosis. The size of Q4 population of HNE1 infected with *BLU* Ad5, and pCD316Ad5, untreated and treated with TRAIL were calculated. The data was presented in **C**. as means ±SD from at least three independent tests. **D**. Drop of mitochondrial membrane potential in TRAIL challenged HNE1 cells. The cells were treated with TRAIL, and stained with TMRE. The drop of mitochondrial membrane potential (MMP) on apoptosis contributed to a depolarized peak left to the main peak of normal MMP. The data from at least three independent tests was summarized in **E**. and presented as mean ± SD.

The microscopic assessment of the PI-stained HNE1 cells’ nucleus, post stimulation with 200 ng/ml TRAIL with 10 μg/mL CHX, revealed substantial apoptosis. The cell number decreased significantly, and the fragmented nuclei were visible (Figure 2A). A death ligand of TNF superfamily, TRAIL, activated two arms of reaction: pro-apoptotic and activation of anti-apoptotic survival supporting the NF-κB pathway. Apoptosis is induced only when the de novo protein synthesis mediated by NF-κB activation is inhibited by CHX. The turnover of the phosphatidylserine to the external membrane is a characteristic of apoptosis. When co-incubated with the designated dose of TRAIL and CHX, the annexin V positive and low PI cell populations increased (in Q4 quadrant); the change was pronounced in BLU-expressing cells (Figure 2B and 2C). The cells are classified into two types based on the mitochondrial involvement to activate the downstream caspases in death receptor-induced apoptosis. The DISC formed on the membrane of type II cells recruits less caspase-8 and weakly triggers the caspase cascade than type I cells. Both the permeabilization of the mitochondrial membrane and the release of a pro-apoptotic factor to cytoplasm occurred in the cells in the current study, indicating a type II phenotype. Disruption of the mitochondrial membrane potential (MMP) is preceded by the alteration of the transition pore of the membrane. As assayed by FACS-based TMRE, the change was rather pronounced in BLU-expressing HNE-1 cells challenged by TRAIL (Figure 2D and 2E).

### TRAIL-induced apoptosis was potentiated in *BLU*-expressing cells, as suggested by promoted cleavage of caspase-8 and PARP by BLU

**Figure 3:**
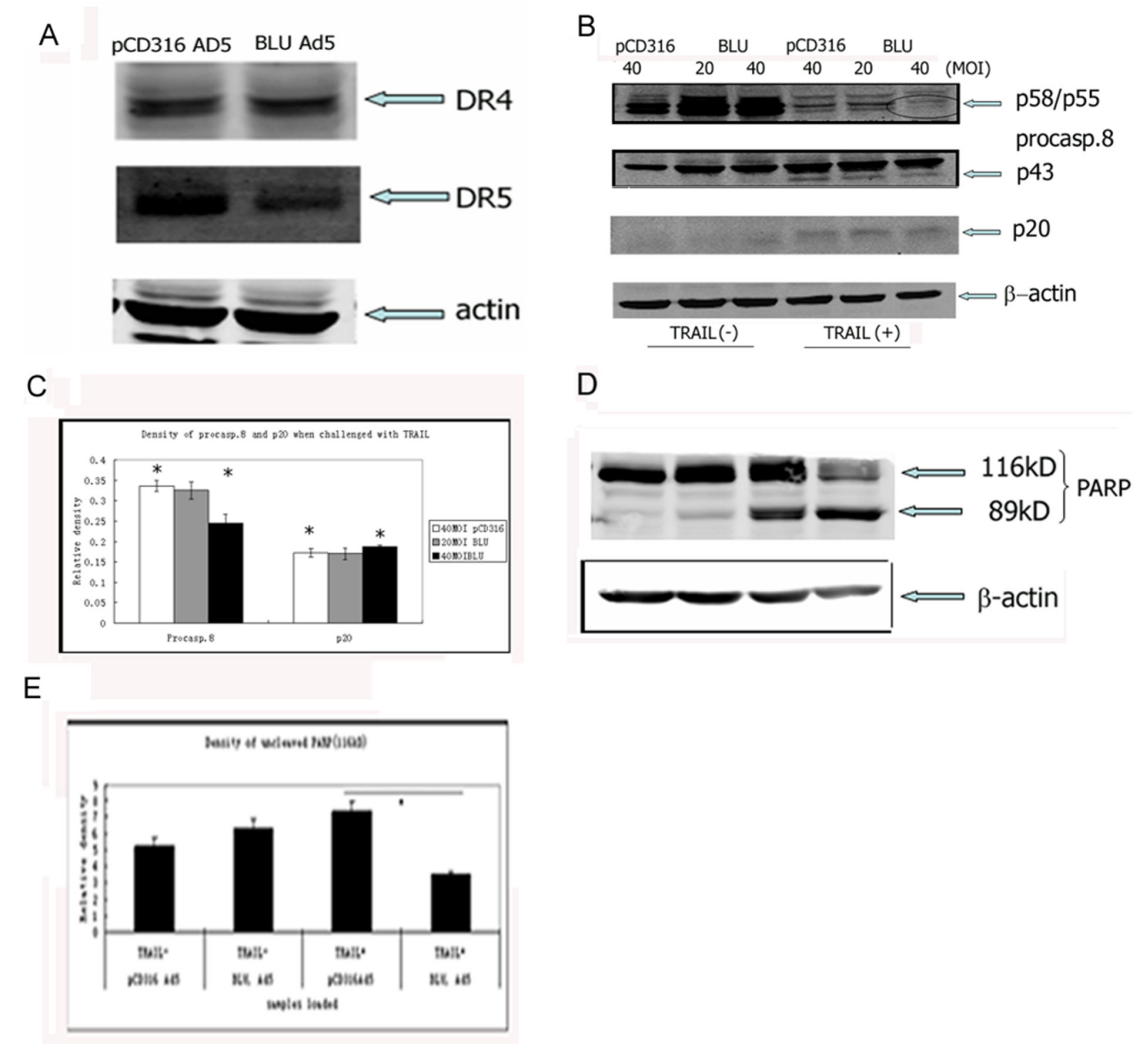
*BLU* promoted TRAIL-induced caspase-8 and PARP degradation **A**. The apoptosis triggering ability of TRAIL in HNE1 cells was confirmed by the expression of death receptor 4 (DR4) and DR5. Total proteins of HNE1 cells infected with pCD316 Ad5 and *BLU* Ad5 were separated by SDS-PAGE, immunoblotted and probed with antibodies, H-130 (against DR4) and D-6 (against DR5). The loading of proteins was calibrated by β-actin (lowest panel). **B**. Cleavage of an initiator caspase, caspase-8, was assayed with total proteins extracted from HNE1 cells infected with pCD316 Ad5 and BLU Ad5, then challenged with TRAIL. They were immunoblotted and probed with specific antibody C15. The cleavage was pronounced in TRAIL treated *BLU* expressing cells. **C**. The relative expression of procaspase-8 to that of β-actin in cells was quantified by a scanning densitometer. The control expression of β-actin was set as 1. **D**. Under similar conditions described in (B), PARP was cleaved. The amount of cleavage of PARP was compared in TRAIL-treated *BLU*-Ad5 and pCD316 Ad5infected HNE1 cells. The weak degradation of PARP in TRAIL-treated *BLU*-Ad5-infected cells suggested that *BLU* was pro-apoptotic. **E**. The relative levels of 116kDa PARP expression to that of β-actin in cells were quantified by a scanning densitometer. The control expression of β-actin was set as 1. The asterisk (*) depicted p<0.05.

The expression of receptors, DR4 or TRAIL R1 and DR5 or TRAIL R2 on HNE1 cells, was present on pCD316-Ad5 or BLU-Ad5 infected HNE1 cells’ surface as detected by immunoblotting (Figure 3A). This expression confirmed that TRAIL possessed the ability to trigger apoptosis in this cell line. During death receptor-induced apoptosis, the aggregated death receptor formed a DISC on the inner surface of the membrane, and the complex recruited initiators such as caspases 8 and 10, catalyzed by autohydrolysis. The zymogen form of caspase-8 was presented as a dimer of isoforms a and b, which were manifested as dimers of 55 and 58kDa, respectively. As a result of co-incubation with TRAIL, 41kDa and 43kDa dimers were generated; the cleavage was remarkable in TRAIL-treated BLU-expressing cells, and additional procaspase-8 was cleaved (Figure 3B). When challenged with TRAIL, PARP was cleaved as shown by the generation of a 95kDa band. Similar to the cleavage of caspase-8, more PARP was degraded in BLU-expressing cells, as indicated by the low amount of the upper band. The data also suggested that cleavage of the apoptotic substrate was promoted by BLU. Although the expression of BLU normally did not trigger remarkable morphological apoptosis, the immunoblot of the BLU-expression showed PARP degradation, a characteristic of apoptosis (Figure 3C). The lanes 1, 2, 3, 4 represented pCD316-Ad5 and BLU-Ad5, untreated with TRAIL; pCD316-Ad5 and BLU-Ad5 treated with TRAIL, respectively.

### Expression of *BLU* downregulated NF-κB signaling by inhibiting reporter activity and reducing the expression IkapakB kinase alpha (IKKα)

**Figure 4:**
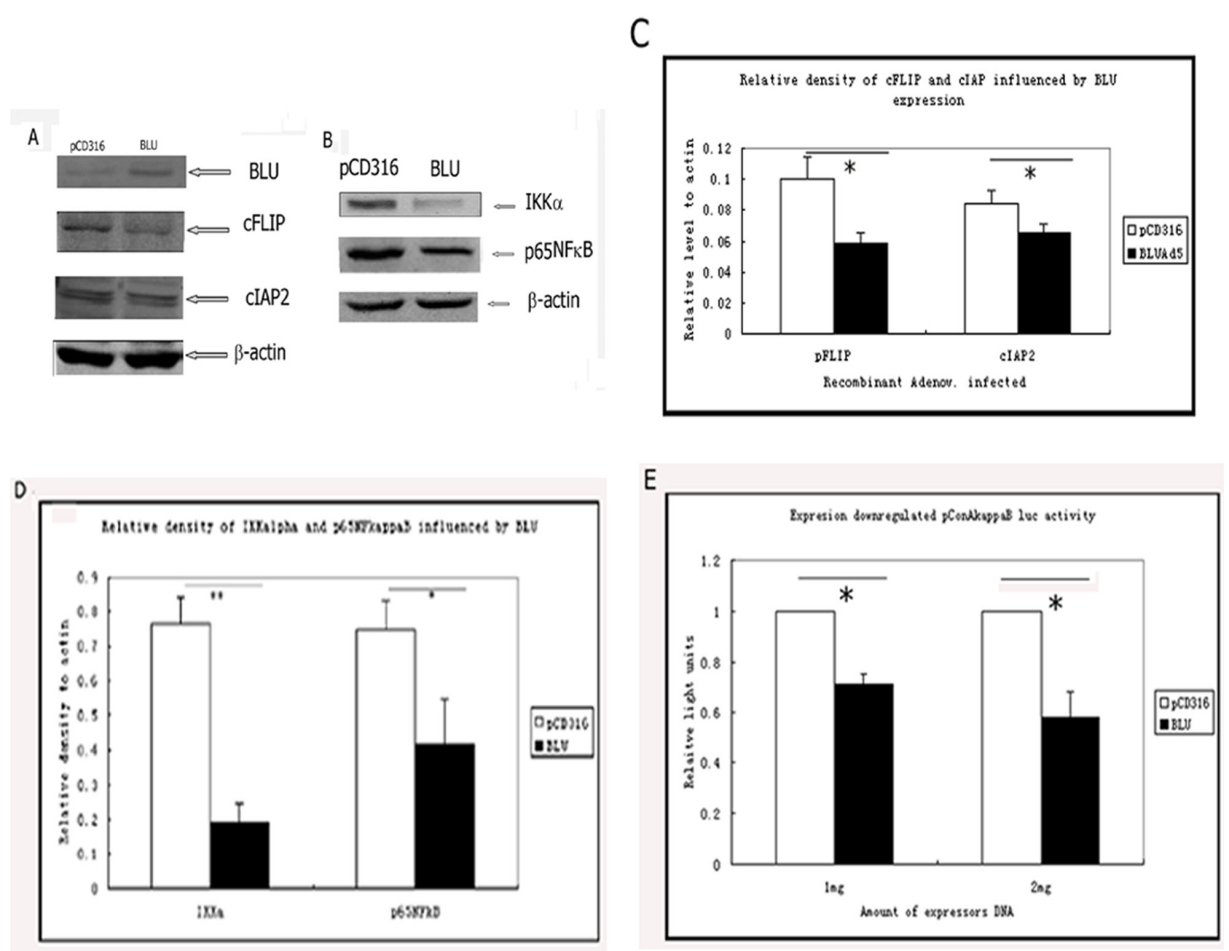
Expression of *BLU*-inhibited signaling of NF-κB and downregulated NF-κB-induced anti-apoptotic factor cFLIP and cIAP2 **A**. Expression of cFLIP_L_ and cIAP2 by HNE1 cells infected with pCD316-AD5 or *BLU*-Ad5. The total proteins from the lysates of cells were immunoblotted and probed with anti-cFLIP_L_ mAb (clone NF6), and anti cIAP2 rabbit polyclonal antibody. The difference of cFLIP and cIAP2 expression level in *BLU*-expressing and control cells was suggested. **B**. The levels of IKKα and p65NF-κB, regulator and an active subunit of NF-κB pathway was evaluated by immunoblotting in BLU-expressing and control cells. The representative data were derived from at least two independent tests. **C**. The relative levels of cFLIP and cIAP2 expression to that of β-actin in cells were quantified by a scanning densitometer. The control expression of β-actin was set as 1. p-values between 40 MOI pCD316 Ad5 and *BLU* Ad5 in comparison to cFLIP and cIAP2 were less than 0.05. **D**. The relative levels of IKKα and p65NF-κB expression to that of actin in cells were quantified by a scanning densitometer. The control expression of β-actin was set as 1. *: p<0.05; **: p<0.01. **E**. Reporter assay of NF-κB binding activity of *BLU*. HNE1 cells were co-transfected with 2μg pCD316 or 2 μg *BLU* pCD316 +0.1 μg pConA-κB-luc, and 1 μg pCD316 or 1 μg BLU pCD316 +0.1 μg pConA-κB-luc, together with renilla luciferase expressor plasmid to calibrate transfection efficiency. The lysate of the cells was mixed with luciferrin and the luciferase activity was recorded by a luminometer. The ratio of the values of firefly (from pConA κB luc.) and renilla luciferase activity was calculated. The values were presented as mean ± SD, derived from at least three independent experiments.

Death receptor-induced apoptosis is regulated by a set of molecules structurally similar to caspase-8, caspase-8-like apoptosis regulator (CLAR), and other anti-apoptotic factors. A well-known member of this family, cFLIP_L_ (cellular FLICE inhibitory protein, long form) is transcription factor NF-κB-dependent. cFLIP_L_ negatively regulates the activity of caspase-8 as a dominant negative binding partner. With the expression of cFLIP_L_, the caspase-8 activity is decreased. Therefore, we examined the level of cFLIP_L_. The data showed that in BLU-expressing HNE1 cells, the expression of cFLIP_L_ was downregulated; also cIAP2 was downregulated in BLU Ad5 in comparison with pCD316 Ad5 infected HNE1 cells (Figure 4A). The quantified data in Figure 3A were presented in Figure 4C. Since the induction of CLAR and cIAP2 is NF-κB-dependent, we tested whether the expression of *BLU* blocked the NF-κB signaling. Additionally, the level of IKKalpha, the component of the complex which catalyzes phosphorylation of I–κ kB and the transcription factor p65 NF-κB, was reduced in *BLU*-expressing HNE-1 cells (Figure 4B, 4D). The pConA- κB-luc reporter containing three κB binding sites revealed low activity when co-transfected to HNE1 cells with *BLU*, or to cells infected with BLU-Ad5 (Figure 4E).

## DISCUSSION

The epigenetic and/or genetic inactivation of TSGs contributes towards the occurrence of malignancy, because of the loss of functions such as inhibition of cell cycle entry and triggering of apoptosis in the host [21, 22]. Such anomaly is implicated in the proliferation of cancer. It has been reported that the re-expression of the frequently lost TSGs restores the normal modulation of cell cycle entry and apoptosis by inhibition of some signaling pathway [32–35]. In the present study, we have shown that re-expression of *BLU*, a TSG mapped on the frequently lost chromosomal region, 3p21, potentiated TRAIL-induced apoptosis.

Antitumor immune surveillance is exerted by two sets of effector cells, CTLs and NK cells [38]. The cells function to eradicate the tumor cells by releasing cytotoxic molecules such as TRAIL and Fas ligand, to stimulate DR-induced or extrinsic apoptosis. The data in the present study suggested that TRAIL possessed the potent ability to kill NPC-derived NHE1 cells. However, various strategies have been shown to be utilized by cancer cells, to evade host immune surveillance and facilitate outgrowth of the malignant clones [39]. Wennerberg et al. reported that an anticancer chemotherapeutic agent, doxorubicin, augmented the activity of NK cells by modulating the signaling TRAIL receptor [40]; the activity of TRAIL is regulated by cancer-related genes and miRNAs [20]. The findings that *BLU*-enhanced apoptosis induced by a chemotherapeutic agent, paclitaxel, prompted to investigate whether *BLU* potentiated DR-induced apoptosis mediated by TRAIL, in order to promote antitumor immune surveillance.

We have shown that infection with *BLU*-expressing recombinant *BLU*-Ad5 remarkably reduced the viability of NPC-derived HNE1 cells, and intratumoral injection of the same recombinant adenovirus inhibited the growth of human NPC xenografts in nude mice. The data further validated the tumor suppressive potential of the *BLU* gene. Functionally, *BLU* may suppress the tumor formation and progression via intracellular pathway targeting. Our previous study indicated that *BLU* downregulated the JNK pathway and inhibited the promoter activity of *CCND1*, coding for cyclin D1, hence arresting the cell cycle in G1 phase [34]. Other studies have shown that *BLU* also downregulated proangiogenic cytokine to inhibit angiogenesis [41]; it also inactivated anti-apoptotic members of the Bcl-2 family to promote paclitaxel-induced apoptosis [35]. In contrast to the findings that forced expression mediated by adenoviral vector was unable to trigger apoptosis in lung cancer-derived cells [42], our study suggested that BLU was pro-apoptotic in HNE1 cells as it induced the cleavage of 116kDa PARP.

Potentiation of TRAIL-induced apoptosis has been observed in our study. Flow cytometry based staining with annexin V, which detects phosphatidylserine (PS) on the outer membrane, revealed only minor difference in apoptotic populations in control cells when infected with *BLU*-Ad5 vs. pCD316 Ad5. However, the apoptotic population was significantly increased in TRAIL-treated *BLU*-expressing cells. Stimulation by TRAIL triggered degradation of an initiator caspase, caspase-8. In HNE1 cells infected with *BLU*-Ad5 and pCD316 Ad5, the procaspase-8 isoforms degraded when challenged with TRAIL. The TRAIL induced cleavage of procaspase-8 was promoted when the cells were infected with 40 MOI *BLU*-Ad5. At this dose, the apoptosis potentiation by *BLU* was suggested.

Caspase-8 presents a differential ability to activate within DISC in order to stimulate the downstream executive caspases like caspase-3, 6, and 7 [43]. Therefore, the cells are classified into two types, depending on whether caspase-8 cleaves a BH3-like Bcl-2 family protein, BID [44, 45] to permeabilize the outer membrane of mitochondria and trigger the release of pro-apoptotic factors, cytochrome C and apoptosis inducing factor (AIF) [46, 47]. In Type II cells, the mitochondria are perturbed, and apoptosomes are formed to activate the caspase cascade, leading to morphological apoptosis [48, 49]. The drop of MMP was observed in HNE1 cells co-incubated with TRAIL, characteristic of the type II cells. The change in the mitochondrial membrane was also pronounced in *BLU*-expressing cells.

DR induced-apoptosis is regulated at the level of DISC, mediated by the ratio of caspase-8 and a structurally similar molecule cFLIP_L_ [50]. cFLIP_L_, of long isoform but not the short isoform c-FLIP_S_, has been shown to be required for caspase-8 activation [51], although the role of the short isoform of FLIP _S_ in inhibition of extrinsic apoptosis could not be entirely excluded [52]. The current study revealed that the expression of *BLU* reduced the level of cFLIP_L_, stimulated the initiator caspase-8, thereby enhanced death receptor-induced apoptosis. Clinicopathologically, the c-FLIP overexpression was correlated with the poor prognosis of NPC [53].

The present study demonstrated that cIAP2, another NF-κB-dependent anti-apoptotic factor [54], which is overexpressed in EBV-associated NPC [55] was downregulated in *BLU*-expressing HNE1 cells. The reduced expression of cFLIP_L_ and cIAP2 by *BLU* led to the hypothesis that tumor suppression by *BLU* was exerted by blocking the NF-κB signaling.

A recently identified tumor suppressor in NPC [56] and esophageal carcinoma, cysteine-rich intestinal protein 2 (CRIP2) encoded by a gene on chromosome 14 has been shown to inhibit NF-κB-dependent proangiogenic cytokine transcription in NPC cells, and its ectopic expression triggers apoptosis in esophageal carcinoma [57].

A tumor suppressor, Von Hippel-Lindau (VHL) on chromosomal 3p25 region, has been shown to suppress NF-κB pathway and enhance TNF-induced apoptosis in renal cell carcinoma (RCC) [58, 59]. The regional deletions on the short arm of chromosome 3 (3p) in RCC is the prototypic alteration in the malignancy-related karyotype of chromosome 3. Additionally, the anomaly of the putative tumor suppressor genes on 3p14, 3p21, and 3p25 is the crucial event in the carcinogenesis of nasopharynx [60, 61]. High expression of inhibitor of differentiation 1 (Id1) and p65 NF-κB have been reported to be correlated with poor prognosis of NPC patients [62].

NF-κB is an anti-apoptotic factor that is implicated in immune response, inflammation, and occurrence of malignancies [11, 15]. During quiescent physiological condition, NF-κB remains inactive because of heterodimerization with an inhibitor subunit I-κB. When I-κB is phosphorylated by upstream kinases, it dissociated from the complex, followed by ubiquitination and degradation. Then, the subunits, such as p65NF-κB, are activated to function as transcription factors [12–14].

The downregulation of the subunit p65 of NF-κB and an upstream kinase IKKalpha caused by the expression of BLU were examined in our study. IKK alpha catalyzes the phosphorylation of I-κB, leading to the activation p65 NF-κB [12]. Though mechanisms remain to be elucidated, the expression of *BLU* reduced the level of IKKα and p65NF-κB and inhibited the activity of NF–κB reporter pConA-κB-luc.

**Figure 5:**
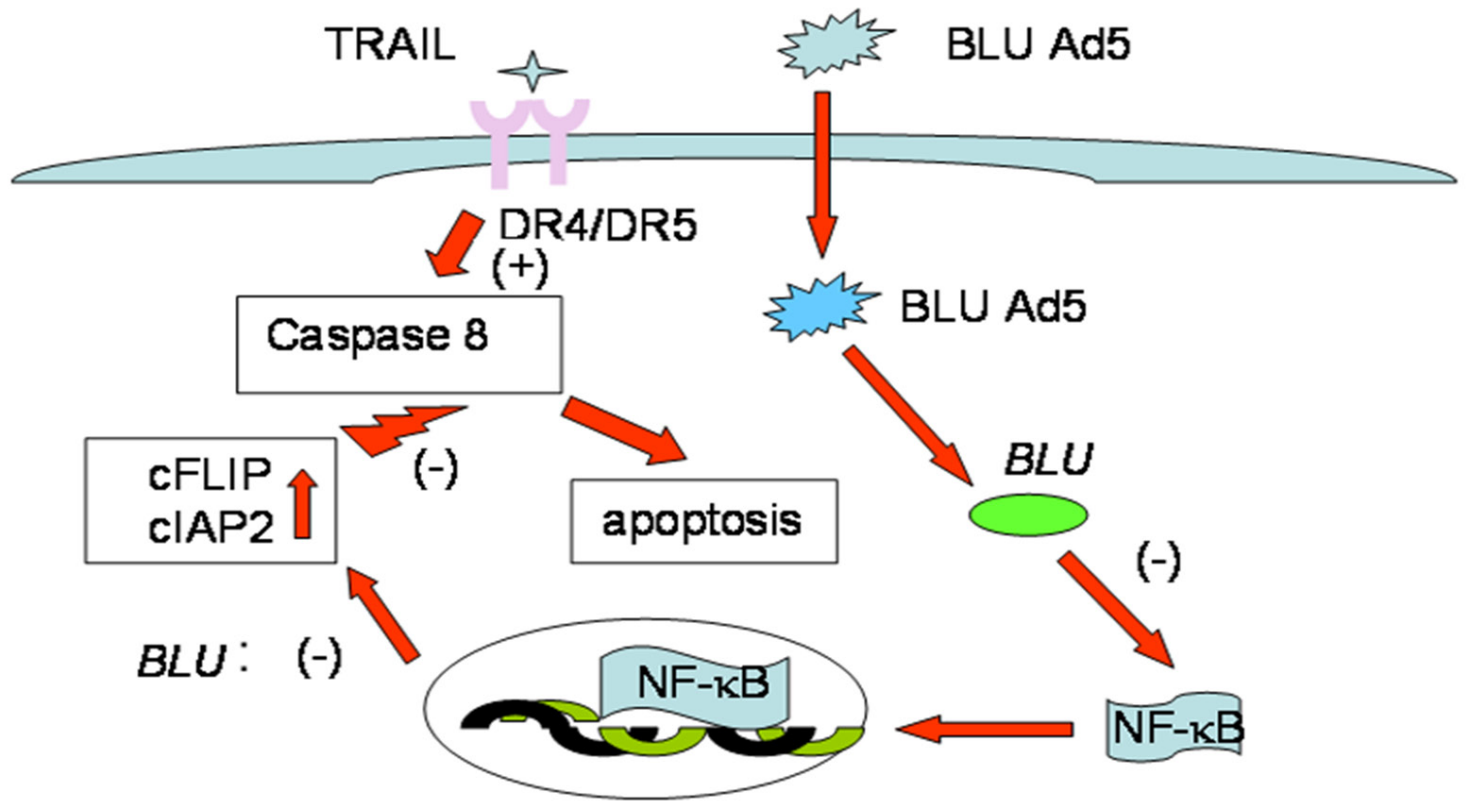
Schematic pattern of Promotion of death receptor induced apoptosis by *BLU* In the present experiment, tumor suppressor BLU which is frequently lost in NPC was ectopically expressed by transfer of adenoviral vector transfer. The expression of *BLU* inhibited signaling of NF-κB hence reduced the expression of anti-apoptotic factors cFLIP and cIAP2. Caspase 8 cleavage triggered by ligation of DR4 and DR5 by TRAIL was enhanced, and apoptosis induced by the two death receptors was promoted and this could contribute to strengthen antitumor immunity.

In immune-deficient individuals, EBV is activated in B lymphocytes to express genomic products with potential of NF-κB induction to transform host cells; the resultant proliferative lymphoid clones progress to lymphoma. Blocking the activated NF-κB with chemical inhibitors such as BAY11-7082 has been promising in treating such disorders [63]. The present study together with other reports [58], suggested NF-κB signaling to be suppressed by a TSG, and it is warranted further validation as a target for anticancer therapy. In summary, promotion of death receptor induced apoptosis by *BLU* through inhibition of NFκB pathway was depicted in Figure 5.

## MATERIALS AND METHODS

### Cells, viral stock, and plasmids

HNE-1 cells derived from undifferentiated nasopharyngeal carcinoma in southern China were a kind gift from Prof. Qian Tao (Chinese University of Hong Kong). They were cultured in DMEM (Gibco Biotechnology, Guangzhou, China) supplemented with 10% fetal calf serum (FCS; Gibco Biotechnology), with or without penicillin + streptomycin (PEST; Gibco Biotechnology), incubated at 37°C with 5% carbon dioxide. Recombinant *BLU* pCD316 plasmid was constructed by cloning a cDNA fragment of BLU in the SacI-SalI site of CMV-derived shuttle vector pCD316. The vector with disrupted E1 and E3 sites was constructed by Dr. Frank Graham [34, 36]. TRAIL with EGFP expression was a kind gift from Dr. Bingliang Fang (MD Anderson Cancer Center) [37]. NF-κB reporter plasmid, pConA-κB-luc, contains three κB binding sites upstream of the coding cassette of luciferase, a kind gift from Prof. Martin Rowe, University of Birmingham, UK.

### Transfection and viral infection

The NPC cells HNE1 were seeded in 6-well plates at a density of 1-2×10^5^cells/ml. Recombinant *BLU*-Ad5 was generated by co-transfection of *BLU*-pCD316 and at least two coding plasmids of the adenoviral capsid protein into packaging cells, with the total amount of 8.5×10^11^ PFU. The cells were then infected with various doses of recombinant *BLU*-Ad5 adenovirus and pCD316-Ad5 for at least 24h after replacing the previous medium. The incorporation of the adenoviral vector was confirmed by fluorescence microscopy and Western blotting. The cells infected with *BLU* and empty vector were challenged with recombinant TRAIL (Pepro Tech, Guangzhou, China). Sterile coverslips were placed on the bottom of the plates. Alternatively, the cells were transfected with TRAIL-EGFP or the empty vector EGFP-N1 utilizing the K2 kit containing Multifier and transfection reagent (Biontex, Martinsried, Germany; a kind gift from Dr. Roland Klösel), according to the manufacturer's protocol. Briefly, the cells were co-incubated with Multiplier for 2h at 37°C, before the DNA and the lipid transfection reagent mixture was gently added to the cells. The expression of the introduced tagged gene was confirmed by fluorescence microscopy. Then, the cells were harvest and fixation with 1:1 acetone-methanol. The cells on the coverslips were stained with 1:100 propidium iodide (PI, Sigma) and screened for apoptotic nuclei induced by TRAIL.

### Cell viability

1×10^5^ cells were seeded in 96-well plates and infected with 0, 5, 10, 20, 40 MOI/cell *BLU* Ad5 adenovirus for 48h, in triplicate wells. The cells were then washed with sterile PBS, CCK8 reagents were added, and co-incubated for 12h, followed by an assessment on a Biotek, plate reader. The mean values and standard deviation (SD) were calculated and represented as mean ± SD derived from at least three independent experiments.

### Luciferase assay of reporter genes

The cells were co-transfected with *BLU* pCD316 or empty vector pCD316, pConA-κB-luc and Renilla Luciferase pGL2 (Promega) with K2 kit according to the manufacturer's instructions. The cells were harvested by lysis with 1x passive lysis buffer (PLB), the lysates collected into microcentrifuge tubes, and the supernatant saved after ultracentrifugation. The luciferase activity was incorporated with a Turner luminometer by mixing the supernatant with luciferin. The relative light activity was presented as the ratio of firefly and renilla luciferase activities. The data was presented as mean ± SD derived from at least three independent tests.

### Western blotting

The cells grown on 6-well plates or Petri dishes were detached by lysis with RIPA buffer containing Triton X-100. The lysates were mixed with 2x Laemmli buffer and DNA strands sheared by sonication. Subsequently, the protein lysates were denatured at 95°C for 5min. The total protein was resolved by polyacrylamide gel electrophoresis (PAGE) and transferred to nitrocellulose membrane. The membrane was blocked with 5% non-fat milk in PBS and then incubated with primary antibodies either at RT for 1h or 4°C overnight. The primary antibodies used in the present study were: rabbit anti-human *BLU* (1:1000, AbCam, Guangzhou, China), murine anti-human actin (1:2000, Sigma, Guangzhou, China), murine anti-human TRAILR1/DR4 (H-130) and TRAILR2/DR5 (D-6) (1:1000, from Santa Cruz, Guangzhou, China). The following antibodies were purchased from Cell Signal Transduction (CST), Guangzhou, China: murine anti-caspase 8, clone C15 (1:500), murine anti-human poly (ADP-ribose) polymerase (PARP) clone C-20 (1:1000), murine anti-IKKα and anti-p65 NF–kapaB (1:1000). The specific murine anti-cFLIP monoclonal antibody, NF6, was a kind gift from Professor Peter Krammer (Program on Tumor Immunogenetics, Deutsches Krebsforschungszentrum, Heidelberg, Germany). After three washes with 0.1% Tween 20 in PBS, the membrane was probed with ultra red labeled anti-murine or rabbit immunoglobulin G (IgG) (Invitrogen, Guangzhou), and the reaction was developed with Odyssey, as previously described [34].

### Apoptosis induction and flow cytometry

The cells were infected with *BLU*-Ad5 or pCD316-Ad5, and challenged by co-incubation with TRAIL+CHX or transfected with TRAIL vector. The cells were harvested, pooled in complete medium, and fixed, followed by sterile PBS washes. Subsequently, the cells were resuspended in PBS, stained using annexin V detection kit, and assessed by FACScan. The cell numbers on quadrant 4, with low PI and high annexin V staining, were considered as apoptotic. Concurrently, the cells with similar parameters were stained with tetramethylrhodamine ethyl ester (TMRE, Immunohistochemistry) and the populations with disrupted mitochondrial membrane potential (MMP), characteristic of apoptosis, were calculated. The data were presented as means ± standard deviation (SD) derived from at least three independent experiments

#### Effect of recombinant *BLU* Ad5 on xenograft in nude mice

The animal breeding conditions and experimental protocols were approved by the medical ethics committee of Guangdong Medical University. Balb/c nude mice of both sexes, aged 4-6 weeks, were purchased from Kunming Institute of Zoology, Chinese Academy of Sciences (CAS). The mice were divided into two groups: *BLU* Ad5 injection and control with pCD316 Ad5. For each group, at least three batches of mice were used, and each batch contained ten mice. The data from at least six mice were used in case of the death of mice during the experiments. 2×10^6^ HNE-1 cells were resuspended in 0.2mL serum-free RPMI, and the suspensions were injected subcutaneously. The palpable tumors were identified in two weeks after inoculation. At this time, 3.5×10^6^ PFU *BLU*-Ad5 was injected intratumorally. Before and after injection, the lengths of the long (a) and short (b) axes were measured with a caliber. The size of the xenograft tumors was measured every two days and allowed to grow for three weeks before harvesting and weighing. Subsequently, the mice were sacrificed by cervical dislocation according to the approved protocols. The tumor size was estimated according to the formula v= ab^2^/2, and that of different periods was presented as means ± SD derived from at least three independent experiments. The growth curves of the treatment and control groups were plotted.

#### Statistical analysis

All the statistical analyses were carried out using the SPSS software program, version 16 (SPSS Inc., Chicago, IL, USA). The quantitative data are presented as means ±SD, derived from three independent tests in a particular assay. P-Values were calculated with Student's t-test, and *p*-values <0.05 were considered statistically significant.
